# 4200 Assessment of differential access to patient online portal (POP) by socioeconomic status (SES) and its impact on asthma care and research

**DOI:** 10.1017/cts.2020.257

**Published:** 2020-07-29

**Authors:** Young J Juhn, Chung-il Wi, Euijung Ryu, Sunghwan Sohn, Miguel Park, Joy Fladager Muth, Hee Yun Seol, Katherine King, Hongfang Liu

**Affiliations:** 1 Mayo Clinic

## Abstract

OBJECTIVES/GOALS: Patient online portal (POP) allows patients to access electronic health records (EHRs) and have efficient communication with their clinicians. We assessed disparities in access to POP by families with different SES and its impact on asthma research which is little known in the literature. METHODS/STUDY POPULATION: A randomized controlled trial testing the efficacy of an EHRs-based clinical decision support (CDS) system was conducted at a pediatric primary care setting of Mayo Clinic. Asthma Control Test (ACT) questionnaire was administered to parents every 3 months through phone or email for this study after consenting, and reminders were sent to unanswered subjects through the POP. SES was measured by HOUSES (in quartiles), a validated individual-level SES index based on housing features (the higher HOUSES, the higher SES).The association of HOUSES with availability of POP access and missing ACT score rate was assessed. RESULTS/ANTICIPATED RESULTS: The mean age of 184 participants was 9.0 years (57% male) and parents of 152 (83%) children had POP. Only 68% of children from lowest HOUSES (Q1) had access to POP (vs. 74% (Q2), 88% (Q3), and 92% (Q4; highest SES); p = .02). ACT score was completed by 144 (78%), 150 (82%), 171 (94%), and 164 (95%) at each intervention conducted every 3 months with a total of 61 (33%) missing at least once. Overall, children whose parents had access to POP had a lower missing rate in ACT score at all interventions during the study; 16% (those with access to POP) vs. 47% (those without), 13% vs. 44%, 3% vs. 16%, and 1% vs. 23% for 1^st^, 2^nd^, 3^rd^, and 4^th^ intervention, respectively (p < .007 for all). DISCUSSION/SIGNIFICANCE OF IMPACT: There are significant disparities in access to POP by SES defined by HOUSES which impact availability of ACT score resulting in a systematic bias in asthma research and potentially widening disparities in asthma care. CONFLICT OF INTEREST DESCRIPTION: NA.

